# Increased Risk of Heart Failure Among Stroke Survivors: A Nationwide Cohort Study

**DOI:** 10.3390/healthcare14121730

**Published:** 2026-06-16

**Authors:** Jung Eun Yoo, Won Hyuk Chang, Bongseong Kim, Heesun Lee, Hea Lim Choi, Junhee Park, Kyungdo Han, Dong Wook Shin

**Affiliations:** 1 Department of Family Medicine, Healthcare System Gangnam Center, Seoul National University Hospital, Seoul 06236, Republic of Korea; 2 Department of Family Medicine, Seoul National University College of Medicine, Seoul 03080, Republic of Korea; 3 Department of Physical & Rehabilitation Medicine, Samsung Medical Center, Sungkyunkwan University School of Medicine, Seoul 06351, Republic of Korea; 4 Department of Statistics and Actuarial Science, Soongsil University, Seoul 06978, Republic of Korea; 5 Division of Cardiology, Department of Internal Medicine, Healthcare System Gangnam Center, Seoul National University Hospital, Seoul 06236, Republic of Korea; 6 Supportive Care Center/Department of Family Medicine, Samsung Medical Center, Sungkyunkwan University School of Medicine, Seoul 06351, Republic of Korea; 7 Department of Clinical Research Design & Evaluation, Samsung Advanced Institute for Health Science & Technology (SAIHST), Sungkyunkwan University, Seoul 06351, Republic of Korea

**Keywords:** stroke, ischemic stroke, hemorrhagic stroke, cardiovascular disease, heart failure

## Abstract

**Highlights:**

**What are the main findings?**

- Stroke survivors, regardless of stroke subtypes, were at significantly greater long-term risk for heart failure than non-stroke controls.
- In particular, stroke survivors with severe disability showed the highest risk of heart failure.

**What are implications of the main findings?**

- Clinicians need to be aware of the importance of heart failure as a long-term consequence of stroke.
- Interventions and targeted prevention strategies addressing modifiable risk factors for heart failure are necessary.

**Abstract:**

**Background/Objectives:** Stroke-induced cardiac damage may lead to lifelong cardiac problems, such as heart failure (HF). We aimed to compare the incidence of HF between stroke survivors and the general non-stroke population. **Methods:** We performed a population-based study of individuals in the Korean National Health Insurance Service database. We included 220,231 stroke survivors between 2010 and 2018 and 1:1 age- and sex-matched non-stroke controls. The study outcome was HF and the cohort was followed up until 2019. Cox hazards models were used to estimate adjusted hazard ratios (aHRs) and 95% confidence intervals (CIs) for HF risk. **Results:** At baseline, stroke survivors had a substantially higher comorbidity burden than matched controls (Charlson Comorbidity Index 4.1 vs. 1.6), with higher rates of hypertension, type 2 diabetes, and dyslipidemia, and were more likely to be current smokers and heavy drinkers. Stroke survivors showed a higher risk for HF (aHR 1.69, 95% CI 1.65–1.73) compared to controls. The risks were further increased with disability, as demonstrated in comparison of those with mild disability (aHR 1.78, 95% CI 1.70–1.87) and those with severe disability (aHR 2.22, 95% CI 2.10–2.36). Before applying the 1-year lag period, the association appeared more prominent among hemorrhagic stroke survivors than ischemic stroke survivors. However, following the 1-year lag period, the HF risk was similar between stroke subtypes (hemorrhagic stroke with disability: aHR 1.77, 95% CI 1.62–1.93; ischemic stroke with disability: aHR 1.71, 95% CI 1.63–1.80). Propensity score matching incorporating all baseline characteristics confirmed the robustness of the primary findings. **Conclusions:** Stroke survivors, who had a greater burden of cardiovascular risk factors, were at significantly greater risk for incident HF compared with age- and sex-matched non-stroke controls, particularly those with disability. Clinicians need to be aware of the importance of HF in stroke survivors who have multiple comorbidities and disability.

## 1. Introduction

Stroke, encompassing both ischemic and hemorrhagic subtypes, remains a leading cause of hospitalization and death worldwide [1,2]. Although advances in acute stroke care and risk factor management have contributed to a substantial decline in early mortality in recent decades, the burden of post-stroke complications continues to pose significant challenges [2]. In particular, new-onset cardiovascular complications have emerged as a major medical concern among stroke survivors [3].

Cardiovascular complications account for approximately one-third of deaths following stroke and substantially contribute to worsening outcomes, reduced quality of life, and increased healthcare-related costs [4]. Evidence from a randomized controlled trial suggests that up to 20% of patients with ischemic stroke develop major cardiac events, ranging from acute myocardial injury and coronary syndromes to HF or arrhythmia, during the acute stroke phase [5]. Even beyond the acute period, the cardiac risk among stroke survivors remains elevated relative to the general population [4].

Stroke-induced cardiac injury may result in potentially lifelong cardiac sequelae, including HF and death [6]. Impaired left ventricular (LV) function has been reported in 8–12% of stroke survivors [7,8,9,10]. However, prior research has predominantly focused on ischemic stroke [7,8,9,10,11] rather than hemorrhagic subtypes, and has largely relied on surrogate markers of HF such as LV systolic dysfunction assessed by echocardiography [7,8,10,12] or elevations in cardiac biomarkers including troponin [7] and brain natriuretic peptide [11,12], rather than clinically confirmed HF diagnoses. Echocardiographic studies may particularly underestimate the true burden of HF, given that HF with preserved ejection fraction accounts for nearly half of all HF cases and may not be captured by assessments of systolic function alone. Furthermore, most prior studies were limited by short follow-up durations, typically within 3 [8,9] or 6 months [11]. Direct comparisons of HF risk between stroke survivors and a non-stroke reference population remain scarce.

Against this background, the present study aimed to investigate the incidence of clinically confirmed HF among stroke survivors relative to the general non-stroke population. We further examined whether the severity of post-stroke disability and stroke subtype (ischemic vs. hemorrhagic) modified the association between stroke and HF risk.

## 2. Materials & Methods

### 2.1. Data Source and Study Setting

This study utilized data from the Korean National Health Insurance Service (NHIS), a government-operated, non-profit single-payer system that covers approximately 97% of the South Korean population. The remaining 3%, comprising the lowest-income individuals, receive coverage through Medical Aid program. The NHIS database encompasses the entire Korean population and includes demographic information, disease diagnosis coded using the International Classification of Diseases, 10th revision (ICD-10), and records of medical treatments and procedures. In addition, the NHIS provides biennial health screenings to all enrolled individuals, during which anthropometric measurements are obtained, and participants complete a self-administered questionnaire covering medical history, current medications, and health behaviors, alongside laboratory test results [13].

### 2.2. Study Population

We initially identified 800,646 stroke cases occurring between 2010 and 2018, defined by the presence of ICD-10 codes I60–I64 during hospitalization, consistent with criteria applied in previous studies [14]. Of these, 335,389 individuals had undergone a health screening within the two years preceding their stroke diagnosis. To ensure analytical validity, we excluded individuals younger than 20 years (*n* = 8), those with a prior diagnosis of HF (*n* = 49,469), myocardial infarction (*n* = 30,377), or atrial fibrillation (*n* = 23,354) before stroke diagnosis, and those with missing data (*n* = 11,950), yielding a final study cohort of 220,231 stroke survivors.

**Figure 1:**
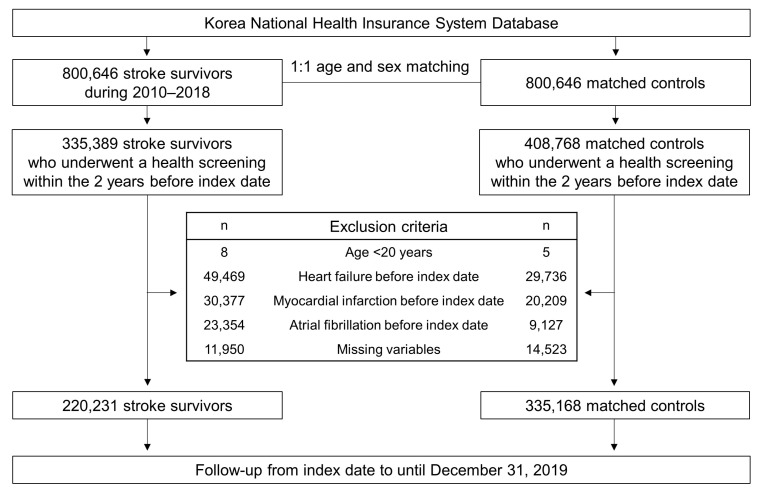
Flow chart of the study population.

For the control group, 1:1 matching by age and sex was performed for each calendar year, such that each stroke survivor was paired with a living control participant of identical age and sex. Controls were assigned an index date corresponding to the stroke diagnosis date of their matched counterpart, and the same exclusion criteria were applied. The process of study population selection is illustrated in Figure 1.

This study was approved by the Institutional Review Board (IRB) of Samsung Medical Center (IRB file no. SMC 2020-12-068). The requirement for informed consent was waived by the review board given that the data were anonymized and publicly available under confidentiality guidelines.

### 2.3. Study Outcomes and Follow-Up

The primary outcome of interest was new-onset HF. Incident HF was defined as the presence of ICD-10 code I50 and at least one hospitalization, a definition widely used in epidemiological studies utilizing claims-based data [15]. Participants were followed from the index date until the first occurrence of HF, death, or the end of the study period on 31 December 2019, whichever came first.

### 2.4. Covariates

Low income was defined as belonging to the lowest 20th percentile of the national income distribution. Residential area was categorized as metropolitan, urban, or rural. Smoking history was classified into three categories: never smoker, former smoker, and current smoker. Alcohol consumption was categorized as none, mild to moderate (less than 30 g per day), or heavy (30 g or more per day). Regular physical activity was defined as engaging in moderate-intensity exercise for more than 30 min on at least five days per week, or vigorous-intensity exercise for more than 20 min on at least three days per week. Body mass index (BMI) was calculated by dividing body weight in kilograms by the square of height in meters. Comorbidities were ascertained from claims data and health screening results obtained prior to the screening date. Hypertension was defined as a diagnosis code of I10–I13 or I15 accompanied by an antihypertensive medication prescription, or a measured systolic/diastolic blood pressure of ≥140/90 mmHg. Type 2 diabetes was defined as a diagnosis code of E11–E14 with an antidiabetic medication prescription, or a fasting glucose level of ≥126 mg/dL. Dyslipidemia was defined as a diagnosis code of E78 with a lipid-lowering medication prescription, or a total cholesterol level of ≥240 mg/dL. Overall comorbidity burden was assessed using the primary care equivalent of the Charlson Comorbidity Index (CCI) [16].

### 2.5. Statistical Analyses

Baseline characteristics of stroke survivors and matched controls were summarized using descriptive statistics. The association between stroke and incident HF was assessed using a Cox proportional hazards model, with results expressed as hazard ratios (HRs) and 95% confidence intervals (CIs). Three models were constructed: Model 1 was an unadjusted model providing crude HRs; Model 2 was adjusted for age, sex, and CCI; and Model 3 further incorporated socioeconomic factors (income level and place of residence), lifestyle variables (smoking, alcohol consumption, and regular physical activity), and comorbidities (hypertension, type 2 diabetes, and dyslipidemia). The proportional hazards assumption was verified using Schoenfeld residuals, and no significant violations were identified.

To examine the influence of stroke severity on HF development, HF risk was assessed according to disability status. Disability grade was determined using the Modified Barthel Index, ranging from 1 to 6 points, with lower scores indicating greater severity. Disability was assessed by specialists in neurology, neurosurgery, or rehabilitation medicine. Participants registered in the National Disability Registry within one year of the index date were classified into disability subgroups, further divided into mild (grades 4–6) and severe (grades 1–3) disability. Additionally, HF risk was analyzed separately according to stroke etiology, distinguishing between ischemic stroke (ICD-10 codes I63–I64) and hemorrhagic stroke (ICD-10 codes I60–I62).

As a sensitivity analysis, we conducted 1:1 propensity score matching (PSM) including age, sex, income level, place of residence, smoking, alcohol consumption, physical activity, BMI, comorbidities, and anthropometric measures as covariates. We performed greedy nearest-neighbor matching without using a caliper. Instead, cases were matched to controls starting from an exact match on the first eight decimal places of the propensity score, progressively relaxing to four decimal places to maximize the number of matched pairs. This digit-based threshold acted as a constraint to ensure sufficiently close matches without applying a fixed caliper width.

All statistical analyses were performed using SAS version 9.4 (SAS Institute, Inc., Cary, NC, USA), and a two-sided *p*-value of less than 0.05 was considered statistically significant.

## 3. Results

### 3.1. Baseline Characteristics

The baseline characteristics of the study population are presented in Table 1. Among the stroke survivors, the mean age was 64.3 (standard deviation, 12.0) years, and 57.8% were male. Compared to matched controls, stroke survivors had lower incomes, were more likely to live in an urban area and to be current smokers and heavy drinkers, and were less likely to engage in regular physical activity. Most metabolic parameters and CCI scores were worse among stroke survivors than their matched controls.

### 3.2. HF Risk Among Stroke Survivors Compared to Matched Controls

**Figure 2:**
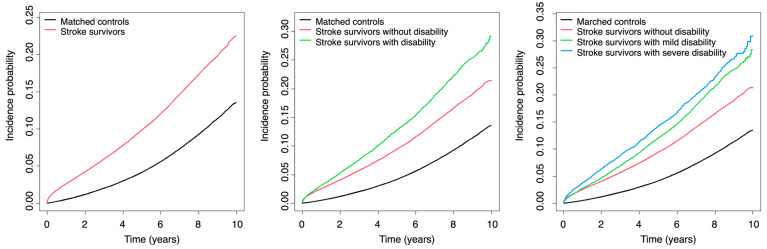
Kaplan–Meier survival analysis for incidence of heart failure in stroke survivors compared to the matched control group.

The mean follow-up durations were 5.3 (standard deviation, 2.5) years and 4.5 (standard deviation, 2.7) years for the matched controls and stroke survivors, respectively. In total, 5.5% of matched controls (18,449/335,168) and 10.0% of stroke survivors (21,990/220,231) developed HF during the follow-up period, resulting in incidence rates of 10.4 and 22.4 per 1000 person-years, respectively (Table 2). Stroke survivors had a higher risk for HF (adjusted HR [aHR], 1.69; 95% CI, 1.65–1.73) compared to the matched controls. Kaplan–Meier curves show that the incidence probabilities of HF in stroke survivors were higher than those in matched control groups (log-rank *p* < 0.001) (Figure 2). The consistent results are noted with a one-year lag (Table 3).

#### 3.2.1. By Stroke Severity

When the risk of HF was analyzed according to the presence of disability, stroke survivors with disability showed a 1.92-fold increased risk for HF (aHR, 1.92; 95% CI, 1.85–1.99) compared to matched controls, which was greater than the risk among those without a disability (aHR, 1.66; 95% CI, 1.62–1.70). There was a dose–response relationship between severity of disability and incident HF: those with mild disability (aHR, 1.78; 95% CI, 1.70–1.87) versus those with severe disability (aHR, 2.22; 95% CI, 2.10–2.36).

#### 3.2.2. By Stroke Type

Stroke survivors displayed an increased risk of incident HF, regardless of the type of stroke. The association seemed to be more prominent in hemorrhagic stroke survivors than ischemic stroke survivors, but, following the one-year lag period, the HF risk by stroke subtype did not differ (e.g., aHR, 1.77 [95% CI, 1.62–1.93] for hemorrhagic stroke with disability and aHR, 1.71 [95% CI, 1.63–1.80] for ischemic stroke with disability).

#### 3.2.3. Sensitivity Analysis

After PSM, 204,910 pairs were included, and all baseline characteristics were well balanced between groups (absolute standardized differences < 0.01 for all) (Table S1). The PSM results were consistent with those of the primary analysis (HR 1.54, 95% CI 1.51–1.57), with a similar dose–response relationship observed according to disability severity, supporting the robustness of the primary findings (Table S2).

### 3.3. Subgroup Analysis

**Figure 3:**
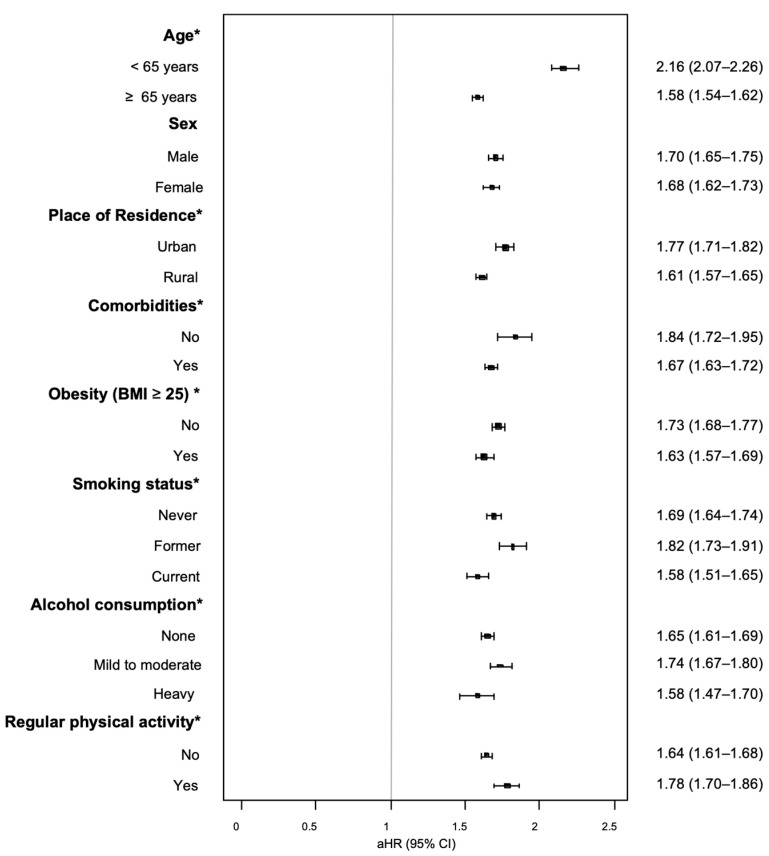
Subgroup analysis for incidence of heart failure in stroke survivors compared to the matched control group. Comorbidities include hypertension, type 2 diabetes, and dyslipidemia; aHR, adjusted hazard ratio; CI, confidence interval; Adjusted for age, sex, Charlson comorbidity index, socioeconomic position (income level and place of residence), lifestyle factors (smoking, alcohol consumption, and regular physical activity), and comorbidities (hypertension, type 2 diabetes, and dyslipidemia). * *p* for interaction < 0.05.

Figure 3 shows the results from stratified analyses by age, sex, place of residence, presence of comorbidities (either hypertension, type 2 diabetes mellitus, or dyslipidemia), obesity, alcohol consumption, smoking, and regular physical activity. Stroke survivors demonstrated a consistently greater incidence of HF in all subgroups compared with the matched controls. The association was more prominent in younger individuals (<65 years), urban residents, those without obesity or comorbidities, mild to moderate drinkers, former smokers, and those who engaged in regular physical activity (*p* for interaction < 0.05 for all).

## 4. Discussion

To the best of our knowledge, this is the first study to directly investigate the relative risk of HF among stroke survivors compared to a general non-stroke control population. The dose–response relationship between disability severity and HF risk, along with consistent findings across various stratified analyses, supports the association between stroke and an increased risk of incident HF. Key strengths of the present study include the use of a nationwide claims database enabling a large sample size, inclusion of age- and sex-matched non-stroke controls with low attrition rates, and the ability to adjust for a wide range of demographic and cardiovascular risk factors through linkage with health screening data.

Acute brain injury, even in the absence of pre-existing cardiac disease, can precipitate cardiac dysfunction, potentially leading to long-lasting cardiac sequelae including HF and death [6]. Shared risk factors for cerebrovascular and cardiovascular disease, such as advancing age, hypertension, type 2 diabetes, and dyslipidemia, may amplify cardiac injury regardless of stroke etiology [6]. Beyond HF, stroke survivors are also at increased risk of other cardiac complications, including atrial fibrillation. Recent nationwide cohort studies using the same Korean NHIS database demonstrated that both ischemic and hemorrhagic stroke survivors were at significantly greater risk of new-onset atrial fibrillation compared to matched controls, further highlighting the broad cardiovascular burden following stroke [17,18]. Consistently, stroke survivors were associated with a 69% increased risk of HF compared with the general non-stroke control population, and this elevated risk was observed regardless of stroke subtype. Therefore, post-stroke HF may be more likely attributable to systemic dysfunction-induced vascular damage, inflammation, and immune responses rather than direct neural causation, though causal relationships cannot be established from the present observational data. It should be noted that these mechanistic pathways were not directly assessed in our dataset and are presented speculatively based on findings from prior experimental and clinical studies.

The pathophysiological mechanisms linking stroke and HF remain incompletely understood, though several pathways have been proposed [19,20]. First, stroke-induced disruption of the hypothalamic–pituitary–adrenal axis and sympathetic hyperactivation trigger a catecholamine surge, resulting in direct catecholamine toxicity, epicardial and microvascular coronary vasoconstriction, and adrenoreceptor-mediated myocardial damage [6]. The topographic distribution of LV β-adrenergic receptors with apical–basal gradients and sympathetic innervation may account for the LV wall motion abnormalities characteristic of neurogenic stress cardiomyopathy [21]. Sustained elevation of circulating catecholamines produces cardiotoxicity [22] and may promote myocardial edema, transient fibrosis, inflammation, and contraction band necrosis [23,24]. Second, neurohormonal dysregulation and systemic inflammation, particularly excessive interleukin-1 release, appear to play a pivotal role in HF development [21]. Pro-inflammatory cytokines generated following brain injury traverse the disrupted blood–brain barrier and enter the systemic circulation, [6] while parasympathetic dysfunction in severe brain injury may further exacerbate myocardial damage through uncontrolled release of inflammatory mediators [25]. The resulting increase in circulating cytokines contributes to a sudden rise in LV afterload and end-systolic pressure [21].

The present findings also demonstrated that HF risk increased proportionally with disability severity. This is consistent with prior evidence showing that cardiovascular risk escalates with the severity of ischemic stroke and the degree of neurological deficits [6]. A higher Hunt–Hess grade on presentation has been identified as a predictor of cardiomyopathy in hemorrhagic stroke [26,27]. Cardiac troponin elevation has been reported in 20–40% of hemorrhagic stroke survivors [28] and 30–60% of those with ischemic stroke [29], and correlates with stroke severity, as well as persistent echocardiographic abnormalities including reduced ejection fraction and regional wall motion abnormalities [30]. Similarly, elevated N-terminal pro-B-type natriuretic peptide (NT-proBNP) levels have been documented among stroke survivors with LV systolic or diastolic dysfunction [26] and have been associated with stroke severity and subsequent cardiovascular events [31].

Stroke survivors demonstrated a consistently greater risk of HF across all subgroups examined. Interestingly, younger stroke survivors had a higher HF risk than older stroke survivors, indicating that unhealthy lifestyle risk factors more prevalent in younger individuals, such as a high body mass index, smoking, and excess alcohol consumption, may contribute to increased risk of HF [32]. In addition, the increase in HF risk was more prominent among those without obesity and other comorbidities like hypertension, type 2 diabetes, and dyslipidemia compared to those with comorbidities. This should be interpreted in the context of their generally lower absolute risk of HF compared to older individuals or those with established cardiovascular risk factors. Subgroup analyses by lifestyle factors revealed that former smokers, mild to moderate drinkers, and those engaging in regular physical activity showed the highest relative risk within their respective categories. These patterns may reflect a combination of ‘sick quitter’ effects, whereby individuals who modified their behaviors due to pre-existing health problems carry higher underlying cardiovascular risk, and competing mortality risk among those with the heaviest lifestyle burden, such as current smokers and heavy drinkers. The more prominent association among urban residents may additionally reflect greater exposure to environmental stressors and sedentary occupational patterns. These findings suggest that stroke itself, independent of background cardiovascular risk factors, may be associated with an increased risk of HF, even in cases where the risk of HF is generally thought to be low.

The present study carries important public health implications by demonstrating that stroke survivors face a substantially elevated long-term risk of incident HF. Although post-stroke LV dysfunction occurs in a considerable proportion of patients, it is often transient and reversible [19]. Cardiac dysfunction emerging during the acute phase of brain injury typically resolves over subsequent weeks in parallel with neurological recovery [33]. For instance, stress-induced cardiomyopathy (e.g., Takotsubo syndrome) secondary to stroke, characterized by transient LV dysfunction with recovery usually within six months, occurs in 15–25% of hemorrhagic stroke survivors but only 0.5–1% of those with ischemic stroke [19]. Massive catecholamine release following subarachnoid hemorrhage is considered the primary mechanism underlying stress-induced cardiomyopathy in this population [34]. Nonetheless, even after excluding incident HF within the first year following stroke, the risk of HF among stroke survivors remained elevated relative to the general non-stroke population, with comparable risk estimates observed between ischemic and hemorrhagic stroke subtypes. These findings reinforce the importance of sustained monitoring and active management of modifiable cardiovascular risk factors among all stroke survivors.

There were several limitations to our study. First, because the database used was not originally designed for stroke research, stroke-specific characteristics, such as infarct size and lesion location, which may influence the degree of autonomic dysregulation and neurogenic cardiac injury, were unavailable. To partially address this gap, we used the disability grade registered in the National Disability Registry as a real-world proxy for stroke severity, which was supported by the observed dose–response relationship between disability severity and HF risk. Second, HF diagnosis was based on claims data and no other assessment tools such as echocardiography. There might be some participants with subclinical HF who could have been missed during detection, and the actual incidence of HF could have been underestimated. Furthermore, our HF definition required hospitalization, which may have led to underestimation of milder or subclinical cases of HF. If all cases were properly included, the observed associations would likely be even more prominent. Additionally, stroke survivors are likely to have more frequent healthcare encounters than matched controls, which may have increased the likelihood of HF detection and introduced surveillance bias. However, the hospitalization-based outcome definition may have partially attenuated this effect. Third, although multivariable adjustment was performed for a range of covariates and matching was restricted to age and sex, stroke survivors had substantially higher baseline cardiovascular risk than matched controls. Therefore, residual confounding from unmeasured variables cannot be fully excluded. Additionally, follow-up duration differed between stroke survivors and matched controls (4.5 vs. 5.3 years), largely reflecting higher competing mortality risk among stroke survivors. This was accounted for in the Cox models using person-time, but residual impact on HF incidence estimates cannot be entirely excluded.

## 5. Conclusions

In conclusion, stroke survivors, who had a greater burden of cardiovascular risk factors, were at significantly greater risk of incident HF compared with age- and sex-matched non-stroke controls, with risk increasing proportionally with post-stroke disability severity. These findings represent an association observed in an observational study and should not be interpreted as evidence of direct causation. Nonetheless, clinicians need to be aware of the importance of HF as a long-term consequence of stroke, especially in survivors with multiple comorbidities and disability, and targeted prevention strategies addressing modifiable risk factors for HF are warranted.

## Figures and Tables

**Table 1:** Baseline characteristics of the study population.

Variables	Matched Controls (*n* = 335,168)	Stroke Survivors (*n* = 220,231)	*p* Value
Age (years)	64.8 ± 12.1	64.3 ± 12.0	<0.001
Sex (male)	195,524 (58.3)	127,387 (57.8)	<0.001
Income (Medicaid + lowest 20%)	63,458 (18.9)	48,124 (21.9)	<0.001
Place of residence (Urban)	146,169 (43.6)	87,742 (39.8)	
Smoking status			
Never	204,833 (61.1)	125,975 (57.2)	<0.001
Former	70,557 (21.1)	35,303 (16.0)	
Current	59,778 (17.8)	58,953 (26.8)	
Alcohol consumption			
None	206,653 (61.7)	135,091 (61.3)	<0.001
Mild to moderate	105,883 (31.6)	66,647 (30.3)	
Heavy	22,632 (6.8)	18,493 (8.4)	
Regular physical activity	73,708 (22.0)	40,096 (18.2)	<0.001
Body mass index (kg/m^2^)	24.0 ± 3.1	24.1 ± 3.2	<0.001
Systolic blood pressure (mmHg)	126.9 ± 15.4	132.0 ± 17.5	<0.001
Diastolic blood pressure (mmHg)	77.3 ± 9.8	80.4 ± 11.34	<0.001
Fasting glucose (mg/dL)	103.1 ± 25.7	110.0 ± 38.1	<0.001
Total cholesterol (mg/dL)	196.0 ± 37.9	200.2 ± 40.4	<0.001
Comorbidities			
Hypertension	161,341 (48.1)	162,453 (73.8)	<0.001
Type 2 diabetes	58,243 (17.4)	63,279 (28.7)	<0.001
Dyslipidemia	111,503 (33.3)	145,295 (66.0)	<0.001
Charlson comorbidity index	1.6 ± 1.8	4.1 ± 2.3	<0.001

Data are presented as number (%) or mean ± standard deviation.

**Table 2:** Hazard ratios and 95% confidence intervals for the incidence of heart failure among stroke survivors compared to the matched control group.

	Subjects (N)	Events (*n*)	Follow-Up Duration (Person-Years)	IR	Model 1 HR (95% CI)	Model 2 HR (95% CI)	Model 3 HR (95% CI)
Matched controls	335,168	18,449	1,770,339.1	10.4	1 (Ref.)	1 (Ref.)	1 (Ref.)
Stroke survivors	220,231	21,990	981,819.7	22.4	2.19 (2.15–2.24)	1.79 (1.75–1.83)	1.69 (1.65–1.73)
**By disability**							
Matched controls	335,168	18,449	1,770,339.1	10.4	1 (Ref.)	1 (Ref.)	1 (Ref.)
Stroke survivors							
No disability	194,519	18,442	860,252.5	21.4	2.10 (2.06–2.14)	1.75 (1.71–1.79)	1.66 (1.62–1.70)
Disability	25,712	3548	121,567.2	29.2	2.86 (2.76–2.96)	2.05 (1.97–2.13)	1.92 (1.85–1.99)
**By severity of disability**							
Matched controls	335,168	18,449	1,770,339.1	10.4	1 (Ref.)	1 (Ref.)	1 (Ref.)
Stroke survivors							
No disability	194,519	18,442	860,252.5	21.4	2.10 (2.06–2.14)	1.75 (1.71–1.79)	1.66 (1.62–1.70)
Mild disability	17,017	2274	81,951.9	27.7	2.71 (2.59–2.83)	1.91 (1.82–1.99)	1.78 (1.70–1.87)
Severe disability	8695	1274	39,615.3	32.2	3.18 (3.00–3.36)	2.38 (2.24–2.52)	2.22 (2.10–2.36)
**By type of stroke**							
Matched controls	335,168	18,449	1,770,339.1	10.4	1 (Ref.)	1 (Ref.)	1 (Ref.)
Stroke survivors without disability							
Hemorrhagic stroke	50,713	3547	203,634.9	17.4	1.70 (1.64–1.76)	1.86 (1.79–1.93)	1.83 (1.76–1.90)
Ischemic stroke	143,806	14,895	656,617.6	22.7	2.23 (2.18–2.27)	1.73 (1.68–1.77)	1.62 (1.57–1.66)
Stroke survivors with disability							
Hemorrhagic stroke	6805	792	32,165.2	24.6	2.40 (2.24–2.58)	2.21 (2.05–2.37)	2.13 (1.98–2.29)
Ischemic stroke	18,907	2756	89,401.9	30.8	3.02 (2.90–3.15)	2.01 (1.93–2.10)	1.86 (1.78–1.94)

IR, incidence rate per 1000 person-years; HR, hazard ratio; CI, confidence interval. Model 1: Unadjusted. Model 2: Adjusted for age, sex, and Charlson comorbidity index. Mode 3: Model 2 + adjusted for socioeconomic position (income level and place of residence), lifestyle factors (smoking, alcohol consumption, and physical activity), and comorbidities (hypertension, type 2 diabetes, and dyslipidemia).

**Table 3:** Hazard ratios and 95% confidence intervals for the incidence of heart failure among stroke survivors compared to the matched control group (1-year lag).

	Subjects (N)	Events (*n*)	Follow-Up Duration (Person-Years)	IR	Model 1 HR (95% CI)	Model 2 HR (95% CI)	Model 3 HR (95% CI)
Matched controls	331,618	16,515	1,430,656.0	11.5	1 (Ref.)	1 (Ref.)	1 (Ref.)
Stroke survivors	191,180	15,739	758,367.6	20.8	1.84 (1.80–1.88)	1.54 (1.50–1.58)	1.48 (1.44–1.51)
**By disability**							
Matched controls	331,618	16,515	1,430,656.0	11.5	1 (Ref.)	1 (Ref.)	1 (Ref.)
Stroke survivors							
No disability	167,801	13,129	664,961.2	19.7	1.75 (1.71–1.79)	1.50 (1.46–1.54)	1.44 (1.40–1.48)
Disability	23,379	2610	93,406.3	27.9	2.47 (2.37–2.58)	1.82 (1.74–1.90)	1.72 (1.65–1.80)
**By severity of disability**							
Matched controls	331,618	16,515	1,430,656.0	11.5	1 (Ref.)	1 (Ref.)	1 (Ref.)
Stroke survivors							
No disability	167,801	13,129	664,961.2	19.7	1.75 (1.71–1.79)	1.50 (1.46–1.54)	1.44 (1.40–1.48)
Mild disability	15,605	1725	63,323.8	27.2	2.40 (2.28–2.52)	1.73 (1.64–1.82)	1.64 (1.56–1.73)
Severe disability	7774	885	30,082.5	29.4	2.63 (2.45–2.81)	2.02 (1.89–2.17)	1.91 (1.79–2.05)
**By type of stroke**							
Matched controls	331,618	16,515	1,430,656.0	11.5	1 (Ref.)	1 (Ref.)	1 (Ref.)
Stroke survivors without disability							
Hemorrhagic stroke	39,593	2277	159,349.6	14.3	1.26 (1.21–1.32)	1.44 (1.38–1.51)	1.42 (1.36–1.49)
Ischemic stroke	128,208	10,852	505,611.6	21.5	1.91 (1.86–1.95)	1.52 (1.47–1.56)	1.44 (1.40–1.48)
Stroke survivors with disability							
Hemorrhagic stroke	6168	537	24,863.9	21.6	1.90 (1.75–2.07)	1.82 (1.66–1.98)	1.77 (1.62–1.93)
Ischemic stroke	17,211	2073	68,542.4	30.2	2.68 (2.56–2.81)	1.82 (1.74–1.91)	1.71 (1.63–1.80)

IR, incidence rate per 1000 person-years; HR, hazard ratio; CI, confidence interval. Model 1: Unadjusted. Model 2: Adjusted for age, sex, and Charlson comorbidity index. Mode 3: Model 2 + adjusted for socioeconomic position (income level and place of residence), lifestyle factors (smoking, alcohol consumption, and physical activity), and comorbidities (hypertension, type 2 diabetes, and dyslipidemia).

## Data Availability

Restrictions apply to the availability of these data. Data were obtained from the Korean National Health Insurance Service and are available https://nhiss.nhis.or.kr/bd/ay/bdaya001iv.do (accessed on 25 February 2026) with the permission of Korean National Health Insurance Service.

## Supplementary Materials

The following supporting information can be downloaded at: https://www.mdpi.com/article/10.3390/healthcare14121730/s1, Table S1. Baseline characteristics of the study population after propensity score matching. Table S2. Hazard ratios and 95% confidence intervals for the incidence of heart failure among stroke survivors compared to the propensity score-matched control group.
