# Supplementary material for: Increased Risk of Heart Failure Among Stroke Survivors: A Nationwide Cohort Study
Source: Healthcare (Basel). 2026 Jun 16;14(12):1730. doi: 10.3390/healthcare14121730 (PMC13299645; doi:10.3390/healthcare14121730)
Supplement: Supplementary file 1 [file healthcare-14-01730-s001.zip › healthcare-4369641-supplementary.pdf]

Table S1. Baseline characteristics of the study population after propensity score matching

Variables	Matched controls (n = 204,910)	Stroke survivors (n = 204,910)	ASD
Age (years)	65.0 ± 12.2	65.0 ± 12.2	0.005
Sex (male)	117,218 (57.2)	117,243 (57.2)	<0.001
Income (Medicaid + lowest 20%)	35,938 (17.5)	35,927 (17.5)	<0.001
Place of residence (Urban)	81,486 (39.8)	81,632 (39.8)	0.001
Smoking status			0.002
Never	122,316 (59.7)	122,167 (59.6)	
Former	35,276 (17.2)	35,270 (17.2)	
Current	47,318 (23.1)	47,473 (23.2)	
Alcohol consumption			0.002
None	128,933 (62.9)	128,745 (62.8)	
Mild to moderate	60,556 (29.6)	60,702 (29.6)	
Heavy	15,421 (7.5)	15,463 (7.6)	
Regular physical activity	38,447 (18.8)	38,438 (18.8)	<0.001
Body mass index (kg/m^2)	24.1 ± 3.1	24.2 ± 3.2	0.003
Systolic blood pressure (mmHg)	130.2 ± 15.8	130.2 ± 16.1	0.001
Diastolic blood pressure (mmHg)	79.2 ± 10.0	79.2 ± 10.4	<0.001
Fasting glucose (mg/dL)	107.1 ± 31.8	107.1 ± 32.6	<0.001
Total cholesterol (mg/dL)	197.9 ± 39.9	198.0 ± 39.1	0.004
Comorbidities			
Hypertension	121,535 (59.3)	122,197 (59.6)	0.007
Type 2 diabetes	47,349 (23.1)	47,608 (23.2)	0.003
Dyslipidemia	79,706 (38.9)	80,060 (39.1)	0.003
Charlson comorbidity index	1.8 ± 1.8	1.8 ±1.8	<0.001

Data are presented as number (%) or mean ± standard deviation

ASD, absolute standardized difference

Table S2. Hazard ratios and 95% confidence intervals for the incidence of heart failure among stroke survivors compared to the propensity score-matched control group

	Subjects (N)	Events (n)	Follow-up duration (person-years)	IR	HR (95% CI)
Matched controls	204,910	15,722	1,054,755.5	14.9	1 (Ref.)
Stroke survivors	204,910	20,481	905,527.7	22.6	1.54 (1.51–1.57)
**By disability**					
Matched controls	204,910	15,722	1,054,755.5	14.9	1 (Ref.)
Stroke survivors					
No disability	180,984	17,197	793,300.8	21.7	1.47 (1.44–1.51)
Disability	23,926	3,284	112,226.9	29.3	1.99 (1.92–2.07)
**By severity of disability**					
Matched controls	204,910	15,722	1,054,755.5	14.9	1 (Ref.)
Stroke survivors					
No disability	180,984	17,197	793,300.8	21.7	1.47 (1.44–1.51)
Mild disability	15,809	2,090	75,610.6	27.6	1.87 (1.79–1.96)
Severe disability	8,117	1,194	36,616.3	32.6	2.23 (2.10–2.37)
**By type of stroke**					
Matched controls	204,910	15,722	1,054,755.5	14.9	1 (Ref.)
Stroke survivors without disability					
Hemorrhagic stroke	47,472	3,325	189,343.7	17.6	1.19 (1.14–1.23)
Ischemic stroke	133,512	13,872	603,957.1	23.0	1.56 (1.53–1.60)
Stroke survivors with disability					
Hemorrhagic stroke	6,336	754	29,698.9	25.4	1.72 (1.60–1.85)
Ischemic stroke	17,590	2,530	82,528.0	30.7	2.09 (2.00–2.18)

IR, incidence rate per 1,000 person-years; HR, hazard ratio; CI, confidence interval
